# A scalable cognitive behavioural program to promote healthy sleep during pregnancy and postpartum periods: protocol of a randomised controlled trial (the SEED project)

**DOI:** 10.1186/s12884-019-2390-8

**Published:** 2019-07-22

**Authors:** Bei Bei, Donna M. Pinnington, Lin Shen, Michelle Blumfield, Sean P. A. Drummond, Louise K. Newman, Rachel Manber

**Affiliations:** 1 Turner Institute for Brain and Mental Health, School of Psychological Sciences, Faculty of Medicine, Nursing and Health Sciences, Monash University, 18 Innovation Walk, Clayton Campus, Victoria 3800 Australia; 2 Centre for Women’s Mental Health, Department of Psychiatry, University of Melbourne, Royal Women’s Hospital, Melbourne, Victoria Australia; 3 Department of Nutrition, Dietetics, Food, School of Clinical Sciences, Faculty of Medicine, Nursing and Health Sciences, Monash University, Clayton, Victoria Australia; 4 Department of Psychiatry and Behavioral Sciences, Stanford University, Palo Alto, CA USA

**Keywords:** Sleep, Insomnia, Pregnancy, Postpartum, Perinatal, Cognitive behavioural therapy

## Abstract

**Background:**

Poor sleep, including symptoms of insomnia are common during pregnancy and postpartum periods. Poor sleep during the perinatal period is linked to impaired daytime functioning, mood disturbance, and risk for chronic insomnia. Cognitive behavioural therapy (CBT) is consistently shown to be efficacious in treating insomnia, but it is largely inaccessible to new mothers, and surprisingly, not part of current perinatal care. This study aims to evaluate the feasibility and efficacy of a scalable CBT-based intervention for better sleep quality.

**Methods:**

In this single-blind randomised controlled trial, eligible nulliparous women are randomised in a 1:1 ratio to either the intervention (CBT) or active control (healthy diet) condition. The interventions are provided from the third trimester till 6 months postpartum. The primary outcome is maternal sleep quality and secondary outcomes are maternal sleep-related impairment, mood, health-related quality of life, relationship satisfaction, and mother-infant-relationship, all assessed using validated instruments at 30- (baseline) and 35 weeks gestation (pregnancy endpoint), and 1.5, 3, and 6 months (postpartum endpoint) after childbirth, with follow-up assessments conducted at 1-year and 2-year postpartum.

**Discussion:**

This study has the potential to address the need for an evidence-based, non-pharmacological sleep intervention tailored for the pregnancy and postpartum periods. The intervention is designed to maximise reach and minimise cost, with the potential to scale up and incorporate in routine perinatal care. With outcomes measured at 8 time points, from the third trimester of pregnancy to 2-year postpartum, this study has the potential to examine both short- and long-term impact on maternal sleep and wellbeing.

**Trial registration:**

ACTRN12616001462471; retrospectively registered on 19/10/2016.

**Electronic supplementary material:**

The online version of this article (10.1186/s12884-019-2390-8) contains supplementary material, which is available to authorized users.

## Background

Sleep disturbance is experienced by the majority of child-bearing women during the pregnancy and postpartum periods [1–3]. The basis of poor sleep during these periods are two-fold. First, physiological changes related to pregnancy and birth-giving (e.g., hormonal changes, fetus growth, increased risk for sleep disordered breathing and restless legs syndrome), and postpartum nighttime infant care are common factors that reduce/disrupt sleep opportunity [4]. Second, by the third trimester, approximately 60% of women experience significant insomnia symptoms with persistent difficulties in initiating and maintaining sleep [5, 6]. Further, for about half of the women with probable insomnia during pregnancy, symptom of insomnia persist into at least two years postpartum [6].

The resulting sleep complaints have been associated with a range of negative mental and physical health outcomes, these include maternal mood disturbance and impaired daytime functioning [7–10], symptoms of, and higher risk for, perinatal depression and anxiety [11, 12], risk of postpartum psychosis in vulnerable women [13], gestational diabetes [14], adverse delivery outcomes such as prolonged labour [15], higher rates of cesarean delivery [15], and preterm birth [16, 17], as well as more near-miss motor vehicle accidents [18].

Integrated behavioural and educational interventions have been consistently shown to improve sleep, and have lasting effects on sleep and wellbeing. Cognitive Behavioral Therapy for Insomnia (CBT-I) is an effective insomnia treatment, and compared to sleep medication alone, its effects are comparable at short-term, and more superior at long-term [19]. Even when environmental factors outside of an individual’s control interfere with sleep, CBT-I based strategies can lead to a significant improvement in sleep. Available evidence shows that CBT-I leads to significant reductions in time awake at night, and insomnia severity when insomnia is comorbid with medical conditions that disrupt sleep [20], such as chronic pain and cancer. One reason CBT-I is effective, even when factors not in one’s control disrupt sleep, is that it increases sleep drive, extinguishes conditioned arousal, and focuses on altering maladaptive behaviours and cognitions that perpetuate poor sleep [21]. When CBT-I was tailored to, and piloted on women with comorbid postpartum depression and insomnia, maternal sleep, depressive symptoms, and fatigue [22] improved significantly. More recently, in-person individual CBT-I is shown in a randomised controlled trial to be effective in treating prenatal Insomnia Disorder [23]. These intervention studies highlight the value of integrating sleep management into perinatal care.

### Aims and hypotheses

Given the high prevalence of sleep disturbance during pregnancy and postpartum periods, there is a critical need for a platform that can be easily integrated into routine perinatal care and is accessible to the broad community. However, expectant mothers are not currently receiving reliable information and evidence-based recommendations to prepare and support them through the significant sleep challenges they face during the perinatal transition. This study adapts and evaluate cognitive behavioural strategies for better sleep in a perinatal population served by an Australian public hospital. Adaptation includes features that allow sustainable integration into routine care, promoting eventual wider dissemination.

The primary aims of this study are to evaluate the feasibility and efficacy of integrating this adaptation in outpatient perinatal care. It is hypothesized that:

1. It will be feasible to integrate such a program in routine perinatal care, and the intervention will be acceptable to patients.
2. Compared to the active control group, the intervention group will report significantly better sleep quality (primary outcome), and secondarily, significantly better sleep-related functioning, mental health, quality of life, relationship with partner, and attachment with infants.

## Methods

### Design

This study utilises a single-blind superiority randomized controlled trial design comparing the intervention to an active control condition, allocated at 1:1 ratio.

### Participants

Participants are 150 expectant mothers enrolled in Childbirth Education at the Royal Women’s Hospital (RWH), a centrally located public hospital specialising in women’s health in Victoria, Australia.

#### Inclusion criteria

- Nulliparas (i.e., not having given birth to older children); only first-time mothers are included as they are the majority of women attending childbirth education, and because testing efficacy in this population could reduce the effects of confounding factors such as the effect of an older child on sleep;
- Age 18 or above;
- Singleton pregnancy;
- Able to read and write in English;
- Have regular access to email and internet.

#### Exclusion criteria

- The use of medications or substance that directly affect sleep (including sleep medications, melatonin, steroid inhalers, antidepressant medications, cannabis, etc.);
- Unstable medical conditions that directly affect sleep;
- Participants who endorse symptoms of, or are known to have any one of the following sleep disorders:
  Sleep apnea: loud snoring, or observed gasping or pauses in breathing, or previously diagnosed with apnea hypopnea index > 15 but not/inadequately treated;
  Previously diagnosed Periodic Limb Movement Disorder with arousal index > 15;
  Restless Legs Syndrome (based on structured interview) occurring 3 times/week, with duration of at least one month and onset prior to pregnancy;
  Circadian Rhythm Sleep-Wake Disorders (based on structured interview). These include irregular and non-24-h sleep-wake types, advanced (if habitual bedtime is earlier than 8 pm and habitual wake time earlier than 4 am, occasional deviation from this schedule allowed) and delayed (if habitual bedtime later than 3 am and habitual wake time later than 11 am, occasional deviation from this schedule allowed) sleep phase types.
- Participants with fixed night shift work between midnight and 5 am, or rotating work schedules that require night shifts during the study period.
- Participants with any of the following mental health conditions as determined by structured clinical interview: Major Depressive Disorder (current); Posttraumatic Stress Disorder (current); Panic Disorder if associated with nocturnal panic attacks > 4 times in the past month; Bipolar Disorder (lifetime); Psychotic Disorders (lifetime); Substance Use Disorders (during pregnancy).

### Procedures

This study is retrospectively registered (ACTRN12616001462471) on 19/10/2016, after the first participant enrolled on 16/5/2016 but before the end of recruitment on 11/01/2017. The delay in registration was due to limited resources during the early phase of recruitment, when recruitment rate was substantially higher than expected. No change in protocol occurred between enrollment of the first participant and protocol registration. The trial is expected to complete by 31/12/2019.

#### Recruitment and consent

Expectant mothers enrolled in singleton pregnancy Childbirth Education classes at the RWH are invited via email to participate in “a research study that aims to evaluate the benefits of two well-being programs for new mothers”. The email invitation contains a link to an online Project Information and Consent Form, and interested participants provide informed consent via a web-based survey form. Online Project Information includes inclusion criteria, project details, and encourages participants to contact the research team with any questions and/or concerns before giving consent to participate. Project information also specifies that participation is confirmed after an initial telephone interview.

#### Telephone interview and screening

After participants provide informed consent, a research assistant assesses further eligibility using a structured interview via telephone. Participants who are excluded due to existing sleep disorder(s) are given contact information of local sleep physicians for further assessment and treatment; those excluded due to a current psychiatric disorder are referred to the Centre for Women’s Mental Health, where women attending the RWH are eligible to receive mental health care.

#### Randomization and blinding

Eligible participants are randomized into either the intervention or a comparison condition using block randomization with random block sizes, which ensure equal sample sizes between groups over time. The randomization plan is generated using an online tool (www.randomization.com), which generates permutations of group assignment in random blocks of 4, 6, and 8, with equal numbers of either condition in each block. Individual participant allocation based on this plan is stored in a database, and only accessed by the research assistant who conducts the interview after eligibility is established. Research assistants who conduct follow-up structured interviews on outcome assessments are blinded to treatment conditions until after completion of the final interview. To avoid accidental breaking of the blind, at each phone contact for outcome assessment, the research assistant asks the participant to refrain from revealing which intervention material they receive.

#### Timing, forms of contact, and participant compensation

Interventions are delivered across six time-points (T1 to T6), and outcomes are measured across seven time points (T1 to T8 but not T3; see Table 1). Therapist-assisted component is delivered via telephone at T1, and as per required via email or telephone between T1 and T6. Written intervention materials are delivered via email using online software (www.mailchimp.com), which automates timing of intervention delivery based on gestational milestones. Assessments are conducted via telephone and online surveys with text message reminders for delayed responses. After completing treatment (T6), participants are invited to re-consent for the one- and two-year follow-up assessments (T7 and T8). Participants receive a voucher of 50 Australian Dollars (AUD; 1 AUD is approximately 0.70 US Dollars) at 2 weeks postpartum (T3), AUD 100 voucher at 6 months postpartum (T6), AUD 50 voucher at 12 months postpartum (T7), and AUD 20 at 24 months postpartum (T8).

**Table 1:** Timing and of Assessments and Intervention

	Pregnancy		Postpartum					
	T1	T2	T3	T4	T5	T6	T7	T8
	30 w	35 w	2 w	1.5 m	3 m	6 m	12 m	24 m
Intervention	X	X	X	X	X	X	–	–
Assessment	X	X	–	X	X	X	X	X

*Note*. Assessment at T1 is conducted immediately before intervention commenced. Assessments at T2, T4 – T6 are conducted 1 week after the delivery of intervention materials. *w* weeks, *m* months

#### Audio and video recording

All telephone interviews and interventions are recorded to assess inter-rater reliability of clinical interviews and treatment fidelity. At T6 and T7, participants are video-recorded during free play with their infants (T6) and strange situation paradigm sessions (T7). Audio/video recordings are stored securely with password protection. Written informed consent from participants are sought for obtaining these audio/video recordings.

### Intervention conditions

All participants receive treatment as usual. If participants require sleep and/or mental health treatment after commencing the trial, they are free to do so. Information about treatments on sleep and mental health is recorded to assist interpretation of findings.

#### The sleep intervention

The sleep intervention aims to address 3 types of perinatal sleep complaints identified in the literature: [1] symptoms of insomnia, [2] pregnancy and infant-related sleep disturbance, and [3] sleep-related daytime impairments (e.g., sleepiness, fatigue). The following evidence-based therapeutic components are included: (a) General skills for better sleep to promote resilience to sleep challenges: sleep hygiene, identifying and addressing unhelpful thoughts and beliefs about sleep, relaxation, and strategies for calming the mind when trying to sleep; (b) Understanding the difference between symptoms of insomnia versus sleep deprivation, and managing sleep initiation and maintenance difficulty using stimulus control; time-in-bed restriction is not included in written materials, but administered by the trained psychologist if necessary; (c) Education about typical sleep patterns of new parents and infants to foster realistic expectations about sleep and normalise some sleep loss; (d) Mindfulness-based strategies targeting physical discomfort, pain, and cognitive arousal; (e) Strategies to promote infant self-soothing to reduce infant awakenings and increase maternal sleep efficiency; (f) Encouragement to prioritise one’s own need for sleep, rest, and self-care, and to use properly timed naps based on sleep and circadian rhythm principles [24]; (g) Skills for managing sleepiness/fatigue; (h) Encouragement to enlist partner and family support.

The delivery of the intervention builds on demonstrated efficacy of therapist-assisted self-help CBT for reducing sleep complaints [25]. Content is delivered via the following 3 means, combined: [1] A 50-min standardised telephone session is delivered by a trained psychologist at the start of the program. The psychologist introduces the rationale underlying recommendations for behavioural changes for optimizing sleep during pregnancy, helps the participant personalise strategies for managing sleep problems using intervention materials, and encourages consistent application of intervention strategies in order to promote sustainable behavioural changes. [2] A series of emails containing text, graphics, and/or audio-based intervention components are delivered at 6 critical milestones as described in Table 1. The intervention materials target sleep challenges specific to each time point (e.g., managing insomnia, physical discomfort, and expectation of postpartum sleep at T1 and T2, managing daytime sleepiness at T3-T5, infant sleep/settling at T2-T6). Each email is designed to be succinct and easy to read on computer, tablet, or phone. [3] Mothers who have difficulty applying the intervention materials can request brief email or telephone clarification from the psychologist.

#### The active control condition

To facilitate interpretation of results, so that observed group differences can be largely attributed to differences in the content of interventions, the control condition needs to control for non-specific effects of attention and participation in a wellbeing program (e.g., contact with health professionals, receiving health information, and expectations of benefit). We therefore presented the study as aiming to promote wellbeing and chose a control intervention, “Healthy Diet”, that [1] has face validity for promoting “perinatal wellbeing”, and [2] is delivered in the same format in terms of method of delivery, timing, frequency, and quantity of information. The “Healthy Diet” intervention provided education on healthy eating habits specific to perinatal milestones. Although diet may indirectly affect sleep, previously reported effects of diet interventions on sleep are small and often inconclusive [26]. Further, the project is promoted as the “SEED” project, which stands for “Sleep, Eat Emotions, and Development”, to further facilitate the face validity of participating in a “wellbeing research”.

Content of the Healthy Diet intervention was adapted from consumer information available to women attending the RWH. It includes the following core components: (a) Nutrients required in the third trimester of pregnancy and before giving birth; (b) Nutrition for breastfeeding; (c) Weight management during the postpartum period; (e) Introducing solid food for the infant; (f) Family eating. The Health Diet intervention is delivered in an identical fashion to the sleep intervention, with a trained dietician performing the same role and functions as the psychologist in the sleep intervention. Specifically, the dietician conducts a 50-min manualised telephone session at the start of either program, and responds to questions via brief emails or telephone if needed. Intervention materials are delivered via automated emails at the same time points as in the sleep intervention (Table 1).

### Measurements

Text below summarises measurements used in this study, and specific details on each measure can be found in the Additional file 1. Table 2 shows when each measure is administered. As different factors contribute to maternal sleep characteristics during pregnancy and postpartum periods, we include 2 primary endpoints: T2 as the pregnancy endpoint, and T6 (immediately post-intervention) as the postpartum endpoint.

**Table 2:** Timing of Measurements

Name of Measure	Pregnancy		Postpartum					
	T1	T2	T3	T4	T5	T6	T7	T8
Structured Interviews								
M.I.N.I. International Neuropsychiatric Interview	X							
Duke Structured Interview for Sleep Disorders	X	X^a^		X^a^	X^a^	X^a^	X^a^	X^a^
Primary Outcomes								
Insomnia Severity Index	X	X		X	X	X	X	X
PROMIS Sleep Disturbance - SF	X	X		X	X	X	X	X
Client Satisfaction Questionnaire, Program Feedback						X		
Secondary Outcomes								
Consensus Sleep Diary (adapted)	X	X		X	X	X	X	X
PROMIS Sleep Related Impairment - SF	X	X		X	X	X	X	X
PROMIS Depression – SF	X	X		X	X	X	X	X
PROMIS Anxiety – SF	X	X		X	X	X	X	X
EQ-5D-5 L	X	X		X	X	X	X	X
Dyadic Adjustment Scale-4	X	X		X	X	X	X	X
Prenatal Attachment Inventory	X	X						
Mother to Infant Bonding Scale				X	X	X	X	X
Adult Attachment Style Questionnaire – Short Form	X						X	
Parental Reflective Functioning Questionnaire							X	X
Emotional Availability Scale (25-min videotaped)						X	^b^	^b^
Strange Situation Procedure (25-min videotaped)							X	
Other Factors								
Ford Insomnia Response to Stress Test	X							
Credibility Expectancy Questionnaire	X							
Demographic and Obstetric Information	X			X		X	X	X
Brief Infant Sleep Questionnaire				X	X	X	X	X
PROMIS Instrumental and Emotional Support – SF	X	X		X	X	X	X	X
Dysfunctional Beliefs and Attitudes about Sleep Scale	X	X		X	X	X	X	X
Glasgow Sleep Effort Scale	X	X		X	X	X	X	X
Reduced Morningness and Eveningness Questionnaire	X	X		X	X	X	X	X
Intervention Adherence and Usefulness		X		X	X	X		
Australian Eating Survey	X					X		
Medical Records Extraction						X		

*Note. X* Measure administered at that time point; *SF* Short form; ^a^Insomnia Module only; ^b^ not administered, but coded from the Strange Situation Procedure

#### Screening

The M.I.N.I. International Neuropsychiatric Interview 7.0 [27] is a short, structured diagnostic interview for DSM 5 psychiatric disorders [28]. The Major Depressive Episode, Manic and Hypomanic Episodes, Substance Use, and the Psychotic Disorders Modules are administered for screening.

The Duke Structured Interview for Sleep Disorders (DSISD) [29] is administered during the screening to rule out sleep disorders, and its Insomnia Module is repeated at all subsequent assessment time points.

#### Outcome measures

Feasibility is assessed using recruitment/dropout rates and email opening rates. Acceptability is assessed using the Client Satisfaction Questionnaire [30], and qualitative feedback from participants.

Efficacy outcomes are assessed with self-report instruments because subjective sleep complaints (and not objectively assessed sleep parameters) share strong association with mental health outcomes [7], and because insomnia is assessed and diagnosed via self-report. We selected instruments with strong psychometric properties.

The primary outcome is maternal sleep quality. Sleep disturbances during the perinatal periods are multifactorial, including symptoms of insomnia, as well as sleep disruptions related to fetus growth and infant care. Therefore, we operationalised the primary outcome into two domains: symptoms of insomnia assessed using Insomnia Severity Index [31], and overall sleep disturbance assessed using PROMIS Sleep Disturbance [32].

Secondary outcomes include Insomnia module on the DSISD [29] for Insomnia Disorder status and modified Consensus Sleep Diary [33] for self-report sleep patterns over the past week (e.g., sleep duration, onset latency, wake after sleep onset, daytime naps). Further secondary outcomes assess the impact of the intervention on maternal wellbeing. These include the following: maternal sleep-related impairment, assessed by PROMIS Sleep Related Impairment [32], maternal mental health, measured with PROMIS Depression and Anxiety [34], maternal health-related quality of life, assessed by EQ-5D-5 L [35], relationship satisfaction via the brief Dyadic Adjustment Scale-4 [36], and mother-infant relationship, measured with the Prenatal Attachment Inventory [37], the Mother to Infant Bonding Scale [38], the Emotional Availability Scales (4th Edition) [39], and the Strange Situation Procedure [40, 41].

#### Other measures

Sample characteristics such as demographics, medical conditions, and obstetric information are collected via self-report at T1 and T4, and via extraction of medical records with participants’ consent.

Infant sleep (Brief Infant Sleep Questionnaire [42]) and social support (PROMIS Instrumental and Emotional Support [43]) will be assessed and examined as covariates. The following constructs will also be measured and explored as predictors and/or mechanisms of treatment outcomes: vulnerability to insomnia under stress, using the Ford Insomnia Response to Stress Test [44], beliefs and attitudes about sleep, using the Dysfunctional Beliefs and Attitudes about Sleep Scale [45], sleep effort, using the Glasgow Sleep Effort Scale [46], chronotype, using the Reduced Morningness Eveningness Questionnaire [47], patients’ perceived credibility and expectancy of treatment, using the Credibility Expectancy Questionnaire [48], general relationship/attachment style, using the Adult Attachment Style Questionnaire – Short Form [49], maternal mentalising and reflective functioning, using the Parental Reflective Functioning Questionnaire [50], and frequency and usefulness of using each intervention component [51].

To promote face validity of the comparison intervention, participants in both groups will also complete the Australian Eating Survey Food Frequency Questionnaire [52], which is of similar length to all sleep measures combined. Physiological factors relevant to dietary intake (e.g., glucose, iron, blood pressure, weight) will be extracted from medical records with participants’ consent. In addition, participants will be asked about infant feeding during the postpartum interviews and this information will be used as a covariate when attachment is assessed as an outcome.

### Ethics considerations

All participants provide informed consent for the participation of themselves and their infants. The consent clarifies that the decision whether or not to participate in the study will not in any way affect usual care, and that participants are free to withdraw from the study at any time. Participants who are excluded due to current psychiatric condition are referred for treatment at the Centre for Women’s Mental Health of the RWH. Any changes in protocol are reported to and approved by the RWH Human Ethics Committee before implemented, and are updated on trial registration.

Adverse events are monitored during each assessment time point by the researcher who conduct the telephone interview. Further, participants are asked to report to the research team immediately if they experience unwanted adverse effects during participation. These events are recorded and included in human research ethics reports.

All data are stored with password protection. Each participant is assigned a numeric code. Personal identifiable information such as names and addresses, are stored separately from the research data. The file linking the numeric code to individual participants is password-protected, and only accessible to individuals listed on the human research ethics application. A data monitoring committee was not used due to limited funding.

Results will be disseminated in peer-reviewed publications, scientific conferences, and via community media outlets (e.g., newspaper articles). If findings are positive, we will seek opportunity to further evaluate the effectiveness of the intervention in clinical settings with the goal of eventual implementation.

### Statistical analyses and power

All analyses will be conducted on an intention-to-treat basis. Missing data is expected in a longitudinal design, and will be addressed using multiple imputation or full information maximum likelihood.

Descriptive statistics will provide information about the feasibility and acceptability of the program (Aim 1), including metrics of recruitment, dropout, email opening rates, patient satisfaction, and qualitative feedback.

To examine group differences in primary and secondary outcomes at each post-baseline time point (Aim 2), multiple regression analyses will be conducted with treatment condition (along with relevant covariates) as the independent variable, and the outcome of interest as the dependent variable. Effect sizes along with 95% confidence intervals will be calculated for the change across time (e.g., pre- vs post-intervention) and for group differences at each time point. The proportion of women meeting diagnostic criteria for Insomnia Disorder, and those above threshold for clinically-significant insomnia symptoms at each time point will also be described.

Changes in outcomes over time in both the overall sample, and the differential change trajectories between treatment conditions will be further explored using mixed effects models. Potential predictors of treatment response will be incorporated as relevant time-invariant and/or time-varying covariates in these models, so their effects on change trajectories can be examined.

Assuming 5% missing data at T1 and 15% at each follow-up [7], the proposed sample size of 150 is powered at 80% (two tailed α = 0.05) to detect a moderate sized difference (*d* = 0.5); moderate to large sizes are expected for primary outcomes [20].

## Discussion

There is a significant gap between treatment availability, service delivery, and the high prevalence of sleep disturbances experienced by women during the pregnancy and postpartum periods. This study is a first step towards filling this gap, by establishing both short- and long-term efficacy of a sleep program tailored to the perinatal population. If successful, this light-weight and scalable intervention holds the potential of being incorporated into routine perinatal care and reach large numbers of women in the community. Midwives/nurses can be trained to incorporate the brief one-on-one support for program materials throughout routine clinical contact.

Further, this project focuses on early intervention and prevention, and takes a proactive approach by providing practical strategies for improving sleep during pregnancy, preparing mothers for significant sleep challenges during the postpartum periods. This has the potential to reduce adverse effects of poor sleep on women and their family.

Finally, sleep medication could lead to dependence in a long-term, and is often avoided by women and their physicians during the perinatal periods. This cognitive behavioural sleep intervention could help increase the awareness of non-pharmacological treatment among healthcare professionals and the public.

## Additional file

Additional file 1: Details of measurements. (DOCX 35 kb)

## Data Availability

Not applicable.

## Abbreviations

CBT: Cognitive behavioural therapy
CBT-I: Cognitive behavioural therapy for insomnia
RWH: Royal Women’s Hospital
SEED: Sleep, eat, emotions, and development (project name)
T1: 30 weeks’ gestation
T2: 35 weeks’ gestation
T3: 2 weeks postpartum
T4: 1.5 months postpartum
T5: 3 months postpartum
T6: 6 months postpartum
T7: 1 year postpartum
T8: 2 years postpartum
