# Supplementary material for: A scalable cognitive behavioural program to promote healthy sleep during pregnancy and postpartum periods: protocol of a randomised controlled trial (the SEED project)
Source: BMC Pregnancy Childbirth. 2019 Jul 22;19:254. doi: 10.1186/s12884-019-2390-8 (PMC6647256; doi:10.1186/s12884-019-2390-8)
Supplement: Supplementary file 1 — Details of measurements. (DOCX 35 kb) [file 12884_2019_2390_MOESM1_ESM.docx]

**Additional file 1: DETAILS OF MEASUREMENTS**

**Title**: A scalable cognitive behavioural program to promote healthy sleep during pregnancy and postpartum periods: Protocol of a randomised controlled trial (the SEED Project).

**Authors**: Bei Bei, Donna M. Pinnington, Lin Shen, Michelle Blumfield, Sean PA Drummond, Louise K Newman, Rachel Manber

## Screening

***The M.I.N.I. International Neuropsychiatric Interview 7.0 (MINI)*** [[1]](https://paperpile.com/c/vElcGa/q88tH) is a short, structured diagnostic interview for DSM-5 psychiatric disorders [[2]](https://paperpile.com/c/vElcGa/Y8hZl) requiring “yes” or “no” responses. It comprises modules, each identified by letters corresponding to diagnostic categories. In this study, the Major Depressive Episode, Posttraumatic Stress Disorder, Panic Disorder, Manic and Hypomanic Episodes, Alcohol Dependence/Abuse, Substance Dependence/Abuse, and the Psychotic Disorders modules will be administered for screening. The MINI is an effective tool for treatment outcome tracking [[1]](https://paperpile.com/c/vElcGa/q88tH), and has been well accepted by patients [[3]](https://paperpile.com/c/vElcGa/LWmI). The MINI has been validated against the SCID-P [[1]](https://paperpile.com/c/vElcGa/q88tH), it has good inter-rater and test-retest reliability, and diagnosis with good to very good sensitivity and specificity [[4]](https://paperpile.com/c/vElcGa/zywB). In this study the MINI will be used to screen for psychiatric disorders as specified in the exclusion criteria.

***The Duke Structured Interview for Sleep Disorders (DSISD)*** [[5]](https://paperpile.com/c/vElcGa/3n5An) is a semi-structured interview that assesses research diagnostic criteria for sleep disorders. A revised version was adapted to bring question content in line with DSM-5 diagnostic criteria [[2]](https://paperpile.com/c/vElcGa/Y8hZl). The DSISD will be used in this study initially to identify women with sleep disorders, and to monitor change, or emergence of insomnia symptoms (in conjunction with the Insomnia Severity Index) in subsequent assessments. The DSISD has demonstrated good reliability and validity [[5]](https://paperpile.com/c/vElcGa/3n5An).

## Primary Outcomes

***The Insomnia Severity Index*** [*[6]*](https://paperpile.com/c/vElcGa/5Mq5x) is a 7-item self-report measure on insomnia symptom severity, sensitive to change in perceived sleep difficulty after intervention. Items such as “difficulty falling asleep”, “difficulty staying asleep” are rated on a 5-point scale (0 = none to 4 = very severe) for the past 2 weeks. Total scores range 0 - 28, with higher scores indicating more severe insomnia symptoms. Specifically, scores of 8-14 indicate subthreshold insomnia, 15-21 moderate clinical insomnia, and 22-28 severe clinical insomnia. High internal consistency (α = .91) was previously reported [[7]](https://paperpile.com/c/vElcGa/poyo).

***The Consensus Sleep Diary*** [[8]](https://paperpile.com/c/vElcGa/IiyUf) was adapted to 7 antenatal, and 10 postpartum questions (e.g., “How long did it take you to fall asleep”) based on participant experience over the past week. The postpartum questions discriminate between time spent awake due to the infant (e.g., “How many minutes earlier than planned did you wake up for reasons related to your baby?”) and time spent spontaneously awake (e.g., “How many minutes earlier than planned did you wake up for reasons NOT related to your baby?”). Some questions require participants to state the number of times (e.g., awake, napping, dozing), as well as the duration of each of those events.

***PROMIS Sleep Disturbance – Short Form (PROMIS-SD-SF)*** [[9]](https://paperpile.com/c/vElcGa/m4xRo). The Patient Reported Outcome Measurement Information System (PROMIS) is a measurement system comprising item-banks developed based on item-response theory to provide highly precise, psychometrically sound, easy-to-use, self-report unidimensional measures across a range of health and well–being domains [[10,11]](https://paperpile.com/c/vElcGa/9Z1M+K0ux). PROMIS scales provide standardized measurement with results reported using a *T*-score, which has a mean of 50 and standard deviation of 10. The PROMIS-SD-SF is an 8-item instrument that assesses sleep disturbance. Participants respond to items such as “I had difficulty falling asleep” on a 5-point scale based on experiences over the past 7 days.

## Secondary Outcomes

The PROMIS Sleep-Related Impairment – Short Form (PROMIS-SRI-SF) [[9]](https://paperpile.com/c/vElcGa/m4xRo) is an 8-item brief measure of wake quality (e.g., daytime sleepiness, functioning). Participants rated items such as “I felt tired” on a 5-point scale ranging from 1 = “Not at all”, to 5 = “Very much”. Higher scores on the scale indicate greater sleep-related impairments.

***PROMIS Depression and Anxiety*** [[12]](https://paperpile.com/c/vElcGa/SD4ib) are brief measures for symptoms of depression and anxiety, with 8 items for each domain. Participants rate items such as “I felt worthless”, “I felt anxious” on a 5-point scale based on their experiences over the past week. Both measures have demonstrated good content validity, and high internal consistency (α > .93).

***EQ-5D-5L*** [[13]](https://paperpile.com/c/vElcGa/fzu0v) is a standardized measure of health-related quality of life comprising two sections: (1) Participants indicate their health status “TODAY” on a 5-point scale across 5 domains : mobility, self-care, usual activities, pain/discomfort, and anxiety/depression. (2) Participants mark how good/bad their health is “TODAY” on a vertical (thermometer style) visual analogue scale from 0 “worst imaginable health state” to 100 “best imaginable health state”. The EQ-5D-5L provides (a) a basic descriptive profile for each domain and a single score relative to a reference group based on age and gender; as well as (b) a single self-reported index value which can be used as a quantitative measure of self-reported health outcome.

***The Dyadic Adjustment Scale -4*** [[14]](https://paperpile.com/c/vElcGa/lFuxg) is a 4-item measure of the degree of couple relationship satisfaction. Items 1, 2, and 3 (e.g., “Do you confide in your mate?”) are rated on a 6-point scale (0 = “all of the time” to 5 = “never”), and item 4 on general satisfaction is rated on a 7-point scale (0 = “extremely unhappy” to 6 = “perfect”). Higher total scores indicate higher relationship satisfaction. The authors of the scale reported comparable psychometric properties to existing brief relationship scales, and the unidimensional construct measured, appeared stable over a 2-year period (Sabourin et al., 2005).

***The Prenatal Attachment Inventory - Revised*** [[15]](https://paperpile.com/c/vElcGa/gve3W) is an 18-item measure of the mother’s attachment to infant during pregnancy with three subscales (6 items each): Anticipation (e.g., “I wonder what the baby looks like now”), Differentiation (e.g., “I know when the baby is asleep”, and Interaction (e.g., “I enjoy feeling the baby move”). Items are rated on a 4-point scale (1 = “almost never” to 5 = “almost always”) based on experiences over the past month. Each subscale has shown adequate internal consistency (α > .70), enabling separate subscale interpretation, or a summated total score. Higher scores indicate higher subjective quality of attachment to unborn infant.

***The Mother to Infant Bonding Scale*** [[16]](https://paperpile.com/c/vElcGa/fVmLQ) is an 8-item rating scale of a mother’s feelings toward her infant. Mothers respond to eight single word adjectives (e.g., “loving”, “protective”) on a 4-point scale (1 = “very much” to 4 = “not at all”). Negative items (e.g., “resentful”) are reversed scored.

***The Emotional Availability Scale (EAS) - 4th edition*** [[17]](https://paperpile.com/c/vElcGa/tsidz) assesses mother-child relationship quality objectively based on a mother–child free play interaction videotaped in a laboratory setting. The EAS assesses the construct of Emotional Availability (EA), defined as “the capacity of a dyad to share an emotionally healthy relationship” [[18]](https://paperpile.com/c/vElcGa/06QR). It employs a multi-dimensional framework to assess the affect and behaviour of both the mother and child. Domains assessed in the mother are Sensitivity, Structuring, Non-intrusiveness, and Non-hostility. Domains assessed in the child are Responsiveness to other and the child’s Involvement of the mother. In this study, room setup is adapted from that used by Nicolson et al. (2013)[[19]](https://paperpile.com/c/vElcGa/EsHD). The protocol involves a 7-minute free-play period between mother and child, followed by a 2-minute separation, then a 3-minute reunion episode [[19]](https://paperpile.com/c/vElcGa/EsHD). See Table S1 for details. The videos are then coded by trained assessors who have undergone stringent rater reliability assessment by the EAS developers [[17]](https://paperpile.com/c/vElcGa/tsidz).

Table S1

*Emotional Availability Scale “free-play” Session at 6 Months Postpartum*

| Stage | Duration | Active Participant/s | Action Description |
| --- | --- | --- | --- |
| 1 | 30 seconds | Mother, Infant, and Experimenter | Observer introduces mother and infant to play room, then leaves. |
| 2 | 20 minutes | Mother and Infant | Mother and infant play freely. After 20 minutes mother leaves the room, as she normally would at home. |
| 3 | ≤2 minutes | Infant | Infant alone on play mat. After 2 minutes, mother returns to the room. |
| 5 | ≥3 minutes | Mother and Infant | Mother greets infant as normally would, joins infant on floor for free play session. |
| 6 | ≤10 minutes | Mother, Infant, and Experimenter | Experimenter enters the play room, and conducts brief questionnaire. |

***Strange Situation Procedure (SSP)*** [[20,21]](https://paperpile.com/c/vElcGa/8CbX+liMm) is the gold standard measure of infant attachment organisation from age 12 to 24 months. It involves the videoing and subsequent objective assessment of a standard sequence of eight episodes that include an unfamiliar environment to the infant (i.e., the presence of a stranger), and the systematic departure and return of the mother (see Table S2). This separation-reunion paradigm is designed to trigger the infant’s attachment and exploration systems, enabling observers to classify infant attachment organisation. The SSP employs a group classification system: “Organized” infants are classified as Secure (Group B), Insecure-Avoidant (Group A), Insecure-Resistant (Group C), whilst “Disorganised” infants are classified as Insecure-Disorganized (Group D) [[21]](https://paperpile.com/c/vElcGa/liMm). Further within-group classification uses subtypes (e.g., Group A1, A2) to further distinguish infants by their observable attachment-behaviours. Observations are coded as infant’s (a) proximity and contact seeking behaviour, (b) contact maintaining behaviour, (c) resistant behaviour; and (d) avoidant behaviour [[22]](https://paperpile.com/c/vElcGa/zQqr). The SSP assessors undergo stringent training and rater reliability assessment.

Table S2

*Adapted Protocol for the Strange Situation Procedures at 12 Months Postpartum*

| Episode | Duration | Active Participant/s | Action Description |
| --- | --- | --- | --- |
| 1 | 30 seconds | Mother, Infant, and Observer | Observer introduces mother and infant to play room, then leaves. |
| 2 | 3 minutes | Mother and Infant | Mother does not participate while infant explores. If necessary, play activities are encouraged after 2 minutes. |
| 3 | 3 minutes | Mother, Infant, and Stranger | Stranger enters. 1^st^ minute: stranger is silent. 2nd minute: stranger converses with mother. 3rd minute: stranger approaches infant. After 3 minutes, mother leaves unobtrusively. |
| 4 | ≤3 minutes | Infant and Stranger | First separation episode. Stranger’s adjusts own behaviour to that of infant. |
| 5 | ≥3 minutes | Mother and Infant | First reunion episode. Mother greets and/or comforts infant, then tries to engage in play. Mother leaves again, saying “bye-bye”. |
| 6 | ≤3 minutes | Infant only | Second separation episode. |
| 7 | ≤3 minutes | Infant and Stranger | Continuation of second separation episode. Stranger enters and adjusts own behaviour to that of infant. |
| 8 | ≥3 minutes | Mother and Infant | Second reunion episode: mother greets, picks up and comforts, then tries to engage in play with infant. |
| EAS | ≥5 minutes | Mother and Infant | Mother joins infant on floor for free play session. |

To reduce participant burden, both the SSP and EAS will be assessed using the SSP protocol at 12 months postpartum, with an additional EAS 5-minute free-play session at the end of the SSP in consultation with EAS developer [[17]](https://paperpile.com/c/vElcGa/tsidz).

All observational assessments are video-recorded using two wall or table-mounted digital cameras with full view of the playroom. One of the two cameras is directed at door to capture infant during separation and reunions, and the other positioned at the rear to capture infant during play/exploration.

## Other Factors

***The Brief Infant Sleep Questionnaire*** [[23]](https://paperpile.com/c/vElcGa/Hrz9U) is a 13-item parent-reported questionnaire of infants’ (0-29 months) sleep in the previous week. Items cover three domains: sleep duration, night awakenings, and method of falling asleep. Parents are also asked whether they consider their child’s sleep as a problem. The scale has been found to correlate with actigraphy and sleep diary measures, and is sensitive to developmental changes in sleep [[24]](https://paperpile.com/c/vElcGa/51c3).

***PROMIS Instrumental and Emotional Support - Short Form*** [[25]](https://paperpile.com/c/vElcGa/XP61H) measures instrumental (4 items; e.g., “do you have someone to run errands if you need it?”) and emotional (4 items; e.g., “I have someone to talk with when I have a bad day”) social support. All items are rated on a 5-point scale (1 = “never” to 5 = “always”). Both scales have high internal consistency (α > .92).

***The Ford Insomnia Response to Stress Test*** [[26]](https://paperpile.com/c/vElcGa/8Vzca) is a 9-item self-report measure of sleep disturbance that is associated with commonly experienced stressful situations. The single construct of “stress-related vulnerability to sleep disturbance” was found to underpin these 9-items. Participants are asked “when you experience the following situations, how likely is it for you to have difficulty sleeping?”. Items (e.g., “after a stressful experience in the evening”) are rated on a 4-point scale (1 = “not likely” to 4 = “very likely”). High scores indicate increased vulnerability to insomnia under perceived stress. The scale has shown to have good internal consistency (α = .83).

***The Dysfunctional Beliefs and Attitudes about Sleep Scale -16*** [[27]](https://paperpile.com/c/vElcGa/VbHFt) is a 16-item measure of sleep-related beliefs and attitudes in insomnia, designed to both evaluate, and monitor change. Items cover 4 domains: (1) perceived consequences of insomnia, (2) worry/helplessness about insomnia, (3) sleep expectations, and (4) medication. Items such as “I need 8 hours of sleep to feel refreshed and function well during the day” are rated on a 0 - 10 scale. Scoring involves taking an overall average, with higher scores indicating greater endorsement of these beliefs. The scale has demonstrated adequate internal consistency (α = .77 - .79) and test-retest (*r* = .83) reliability.

***The Glasgow Sleep Effort Scale*** [[28]](https://paperpile.com/c/vElcGa/UQjIC) is a 7-item self-report measure of sleep effort. Participants rate items (e.g., “I feel I should be able to control my sleep”) on a 3-point scale based on experiences over the past week, with higher scores indicating higher sleep effort. The scale has adequate internal consistency (α = .77), and has been shown to reliably discriminate insomnia patients from good sleepers using a cut-off score of 2 [[28]](https://paperpile.com/c/vElcGa/UQjIC).

***Reduced Morningness and Eveningness Questionnaire*** [[29]](https://paperpile.com/c/vElcGa/srgu) is a 5-item scale that assesses chronotype (i.e., Morningness-Eveningness), an individual’s preferred timing for mental and physical activities. It is an abbreviated version of the original 19-tem scale [[30]](https://paperpile.com/c/vElcGa/CTj6), and has shown to correlate positively with the full scale. Each item is scored separately, and combined to form a total score, with higher scores indicating greater morning chronotype and lower scores indicate greater evening chronotype.

***The Credibility Expectancy Questionnaire*** [[31]](https://paperpile.com/c/vElcGa/qJALm) is a 6-item measure of perceived “Credibility” (i.e., how believable, convincing, and logical a treatment is to the patient) and Expectancy” (i.e., the improvements the patient believes will occur after treatment). Items are rated on either a 9- or 10-point scale, with higher total scores reflecting greater credibility and expectancy by the participant”. Internal consistency is good for “Credibility” (α = .81 - .86), “Expectancy” (α = .79 - .90), and for the overall scale (α = .84 - .85).

***The Attachment Styles Questionnaire - Short Form*** [[32]](https://paperpile.com/c/vElcGa/xgMm) is a 15-item measure of general relationship/attachment style. Items are rated on a 5-point scale and summed to form 4 separate attachment style scales: secure, fearful, preoccupied, and dismissing.

***Parental Reflective Functioning Questionnaire*** [[33]](https://paperpile.com/c/vElcGa/h2JE) is a validated, brief, self-report measure that assesses parental reflective functioning, which is also referred to as mentalising, or the ability to “hold others’ minds in mind” [[34]](https://paperpile.com/c/vElcGa/gVtr). In this study, the scale measures the mother’s capacity to reflect on her own internal mental experiences as well as those of her infant. This capacity develops within the context of early attachment relationships, and this measure will provide information on the mother’s internal mentalizing process in the context of observational measures of the mother-infant relationship assessed using EAS and SSP.

***Intervention Adherence and Helpfulness.*** At T2, T4-T6, Participants are asked, how useful new information (i.e., knowledge) they received are, how often they have applied the strategies over the past week, and how useful the strategies have been. These items will be rated on a 0 to 4 scale, with 0 being “not at all useful (for usefulness)” or “never (for frequency)”, and 4 being “very useful (for usefulness)”, and “6-7 days/week (for frequency)”. In addition, participants are asked to describe what challenges (if any) they encountered when applying the strategies. Both the treatment and control conditions receive this scale in the same format, with content for either based on core components of the respective intervention.

***The Australian Eating Survey Food Frequency Questionnaire*** [[35]](https://paperpile.com/c/vElcGa/NueBA) is a semi-quantitative online food frequency questionnaire that assesses eating behaviours and usual dietary/nutritional intake, including energy, protein, fat, saturated fat, carbohydrate, alcohol, fibre, as well as 12 nutrients: thiamin, niacin, riboflavin, folate, calcium, vitamin A, phosphorous, zinc, iron, magnesium, sodium and potassium. This 120-item scale takes approximately 15 minutes to complete, and generates a personalised report on nutritional adequacy of dietary intake tailored to age, gender and life stage.

## Program Evaluation

***The Client Satisfaction Questionnaire*** [[36]](https://paperpile.com/c/vElcGa/uHSey) is an 8-item scale that assesses overall satisfaction with intervention upon study completion. Items such as “to what extent has our program met your needs” are rated on a 4-point scale, ranging from 1 – 4, with a total score ranging from 8 (low satisfaction) to 32 (high satisfaction). The scale has previous demonstrated high internal consistency, with Cronbach's α = .93.

***Qualitative Feedback.*** In addition, at the end of the program, participants are asked to elaborate what they liked about the program, aspects that can be improved, and any other comments they might have.

# References

1 [Sheehan DV, Lecrubier Y, Harnett Sheehan K, *et al.* The validity of the Mini International Neuropsychiatric Interview (MINI) according to the SCID-P and its reliability. *Eur Psychiatry* 1997;**12**:232–41.](http://paperpile.com/b/vElcGa/q88tH)

2 [American Psychiatric Association. *Diagnostic and Statistical Manual of Mental Disorders (DSM-5®)*. American Psychiatric Pub 2013.](http://paperpile.com/b/vElcGa/Y8hZl)

3 [Pinninti NR, Madison H, Musser E, *et al.* MINI International Neuropsychiatric Schedule: clinical utility and patient acceptance. *Eur Psychiatry* 2003;**18**:361–4.](http://paperpile.com/b/vElcGa/LWmI)

4 [Lecrubier Y, Sheehan DV, Weiller E, *et al.* The Mini International Neuropsychiatric Interview (MINI). A short diagnostic structured interview: reliability and validity according to the CIDI. *Eur Psychiatry* 1997;**12**:224–31.](http://paperpile.com/b/vElcGa/zywB)

5 [Edinger JD, Wyatt JK, Olsen MK, *et al.* Reliability and validity of the Duke Structured Interview for Sleep Disorders for insomnia screening. In: *Sleep*. AMER ACAD SLEEP MEDICINE ONE WESTBROOK CORPORATE CTR, STE 920, WESTCHESTER, IL 60154 USA 2009. A265–A265.](http://paperpile.com/b/vElcGa/3n5An)

6 [Bastien CH, Vallières A, Morin CM. Validation of the Insomnia Severity Index as an outcome measure for insomnia research. *Sleep Med* 2001;**2**:297–307.](http://paperpile.com/b/vElcGa/5Mq5x)

7 [Morin CM, Belleville G, Bélanger L, *et al.* The Insomnia Severity Index: psychometric indicators to detect insomnia cases and evaluate treatment response. *Sleep* 2011;**34**:601–8.](http://paperpile.com/b/vElcGa/poyo)

8 [Carney CE, Buysse DJ, Ancoli-Israel S, *et al.* The consensus sleep diary: standardizing prospective sleep self-monitoring. *Sleep*](http://paperpile.com/b/vElcGa/IiyUf) 2012;35(2):287-302.

9 [Yu L, Buysse DJ, Germain A, *et al.* Development of short forms from the PROMIS^TM^ sleep disturbance and Sleep-Related Impairment item banks. *Behav Sleep Med* 2011;**10**:6–24.](http://paperpile.com/b/vElcGa/m4xRo)

10 [Cella D, Yount S, Rothrock N, *et al.* The Patient-Reported Outcomes Measurement Information System (PROMIS): progress of an NIH Roadmap cooperative group during its first two years. *Med Care* 2007;**45**:S3–11.](http://paperpile.com/b/vElcGa/9Z1M)

11 [Cella D, Riley W, Stone A, *et al.* The Patient-Reported Outcomes Measurement Information System (PROMIS) developed and tested its first wave of adult self-reported health outcome item banks: 2005--2008. *J Clin Epidemiol* 2010;**63**:1179–94.](http://paperpile.com/b/vElcGa/K0ux)

12 [Pilkonis PA, Choi SW, Reise SP, *et al.* Item banks for measuring emotional distress from the Patient-Reported Outcomes Measurement Information System (PROMIS®): depression, anxiety, and anger. *Assessment* 2011;**18**:263–83.](http://paperpile.com/b/vElcGa/SD4ib)

13 [Herdman M, Gudex C, Lloyd A, *et al.* Development and preliminary testing of the new five-level version of EQ-5D (EQ-5D-5L). *Qual Life Res* 2011;**20**:1727–36.](http://paperpile.com/b/vElcGa/fzu0v)

14 [Sabourin S, Valois P, Lussier Y. Development and validation of a brief version of the dyadic adjustment scale with a nonparametric item analysis model. *Psychol Assess* 2005;**17**:15–27.](http://paperpile.com/b/vElcGa/lFuxg)

15 [Pallant JF, Haines HM, Hildingsson I, *et al.* Psychometric evaluation and refinement of the Prenatal Attachment Inventory. *J Reprod Infant Psychol* 2014;**32**:112–25.](http://paperpile.com/b/vElcGa/gve3W)

16 [Taylor A, Atkins R, Kumar R, *et al.* A new Mother-to-Infant Bonding Scale: links with early maternal mood. *Arch Womens Ment Health* 2005;**8**:45–51.](http://paperpile.com/b/vElcGa/fVmLQ)

17 [Biringen Z. Emotional availability (EA) scales manual: Part 1. Infancy/early childhood version (child aged 0--5 years). *Unpublished manuscript* 2008.](http://paperpile.com/b/vElcGa/tsidz)

18 [Biringen Z, Derscheid D, Vliegen N, *et al.* Emotional availability (EA): Theoretical background, empirical research using the EA Scales, and clinical applications. *Dev Rev* 2014;**34**:114–67.](http://paperpile.com/b/vElcGa/06QR)

19 [Nicolson S, Judd F, Thomson-Salo F, *et al.* Supporting the adolescent mother-infant relationship: preliminary trial of a brief perinatal attachment intervention. *Arch Womens Ment Health* 2013;**16**:511–20.](http://paperpile.com/b/vElcGa/EsHD)

20 [Ainsworth MD, Bell SM. Attachment, exploration, and separation: illustrated by the behavior of one-year-olds in a strange situation. *Child Dev* 1970;**41**:49–67.](http://paperpile.com/b/vElcGa/8CbX)

21 [Main M, Solomon J. Discovery of an insecure-disorganized/disoriented attachment pattern. In: Brazelton TB, Yogman MW, eds. *Affective development in infancy*. Westport, CT: Ablex Publishing 1986. 95–124.](http://paperpile.com/b/vElcGa/liMm)

22 [Ainsworth MDS, Blehar MC, Waters E, *et al.* *Patterns of attachment: A psychological study of the strange situation*. Psychology Press 2015.](http://paperpile.com/b/vElcGa/zQqr)

23 [Sadeh A. A brief screening questionnaire for infant sleep problems: validation and findings for an Internet sample. *Pediatrics* 2004;**113**:e570–7.](http://paperpile.com/b/vElcGa/Hrz9U)

24 [Mindell JA, Li AM, Sadeh A, *et al.* Bedtime routines for young children: a dose-dependent association with sleep outcomes. *Sleep* 2015;**38**:717–22.](http://paperpile.com/b/vElcGa/51c3)

25 [Hahn EA, DeWalt DA, Bode RK, *et al.* New English and Spanish social health measures will facilitate evaluating health determinants. *Health Psychol* 2014;**33**:490–9.](http://paperpile.com/b/vElcGa/XP61H)

26 [Drake C, Richardson G, Roehrs T, *et al.* Vulnerability to stress-related sleep disturbance and hyperarousal. *Sleep* 2004;**27**:285–91.](http://paperpile.com/b/vElcGa/8Vzca)

27 [Morin CM, Vallières A, Ivers H. Dysfunctional beliefs and attitudes about sleep (DBAS): validation of a brief version (DBAS-16). *Sleep* 2007;**30**:1547–54.](http://paperpile.com/b/vElcGa/VbHFt)

28 [Broomfield NM, Espie CA. Towards a valid, reliable measure of sleep effort. *J Sleep Res* 2005;**14**:401–7.](http://paperpile.com/b/vElcGa/UQjIC)

29 [Adan A, Almirall H. Horne & Östberg morningness-eveningness questionnaire: A reduced scale. *Pers Individ Dif* 1991;**12**:241–53.](http://paperpile.com/b/vElcGa/srgu)

30 [Horne JA, Ӧstberg O. Morningness-Eveningness Questionnaire. PsycTESTS Dataset. 1976. doi:](http://paperpile.com/b/vElcGa/CTj6)[10.1037/t02254-000](http://dx.doi.org/10.1037/t02254-000)

31 [Devilly GJ, Borkovec TD. Psychometric properties of the credibility/expectancy questionnaire. *J Behav Ther Exp Psychiatry* 2000;**31**:73–86.](http://paperpile.com/b/vElcGa/qJALm)

32 [Chui W-Y, Leung M-T. Adult attachment internal working model of self and other in Chinese culture: Measured by the Attachment Style Questionnaire—Short Form (ASQ-SF) by confirmatory factor analysis (CFA) and item response theory (IRT). *Pers Individ Dif* 2016;**96**:55–64.](http://paperpile.com/b/vElcGa/xgMm)

33 [Luyten P, Nijssens L, Fonagy P, *et al.* Parental Reflective Functioning: Theory, Research, and Clinical Applications. *Psychoanal Study Child* 2017;**70**:174–99.](http://paperpile.com/b/vElcGa/h2JE)

34 [Fonagy P, Gergely G, Jurist E, *et al.* *Affect regulation, mentalization, and the development of the self*. New York: Other Press 2002.](http://paperpile.com/b/vElcGa/gVtr)

35 [Collins CE, Burrows TL, Rollo ME, *et al.* The comparative validity and reproducibility of a diet quality index for adults: the Australian Recommended Food Score. *Nutrients* 2015;**7**:785–98.](http://paperpile.com/b/vElcGa/NueBA)

36 [Attkisson CC, Zwick R. The client satisfaction questionnaire. Psychometric properties and correlations with service utilization and psychotherapy outcome. *Eval Program Plann* 1982;**5**:233–7.](http://paperpile.com/b/vElcGa/uHSey)
